# A cross-sectional study of the environment, physical activity, and screen time among young children and their parents

**DOI:** 10.1186/1471-2458-14-61

**Published:** 2014-01-21

**Authors:** Valerie Carson, Andrei Rosu, Ian Janssen

**Affiliations:** 1 Faculty of Physical Education and Recreation, University of Alberta, W1-34 Van Vliet Centre, Edmonton, AB T6G 2H9, Canada; 2 School of Kinesiology and Health Studies, Queen’s University, 28 Division St., Kingston, ON K7L 3N6, Canada; 3 Department of Community Health and Epidemiology, Queen’s University, Carruthers Hall, Kingston, ON K7L 3N6, Canada

**Keywords:** Physical activity, Screen time, Physical environment, Child, Preschool, Parents

## Abstract

**Background:**

To develop evidence-based interventions promoting healthy active lifestyles among young children and their parents, a greater understanding is needed of the correlates of physical activity and screen time in these dyads. Physical environment features within neighborhoods may have important influences on both children and their parents. The purpose of this study was to examine the associations between several features of the physical environment with physical activity and screen time among 511 young children (≤5 years old) and their parents, after adjusting for socio-demographic factors.

**Methods:**

From May to September, 2011, parents of 0–5 year old children from Kingston, Canada completed a questionnaire that assessed socio-demographic characteristics, their physical activity and screen time, and their child’s physical activity and screen time. Guided by a previously developed conceptual framework, several physical environment features were assessed using Geographic Information Systems including, function (walkability), safety (road speed), aesthetics (streetscape), and destination (outdoor play/activity space, recreation facilities, distance to closest park, yard at home). Multilevel linear regression analyses were used to examine the relationships while adjusting for several socio-demographic factors.

**Results:**

The only independent association observed for the physical environment features was between higher outdoor play/activity space and higher screen time levels among parents. Several associations were observed with socio-demographic variables. For physical activity, child age, child care status, and family socioeconomic status (SES) were independent correlates for children while sex was an independent correlate for parents. For screen time, child age and family SES were independent correlates for children while neighborhood SES was an independent correlate for parents.

**Conclusions:**

The findings suggest that socio-demographic factors, including social environment factors, may be more important targets than features of the physical environment for future interventions aiming to promote healthy active lifestyles in young children and their parents. Given this was one of the first studies to examine these associations in young child–parent dyads, future research should confirm and build on these findings.

## Background

Regular physical activity is associated with numerous health benefits among young children [1] and their parents [2]. For example, physical activity is positively associated with motor skill development and psychosocial health and negatively associated with adiposity and cardio-metabolic risk factors among 0- to 4-year-olds [1]. Similarly, a dose–response relationship between physical activity, several chronic conditions (e.g., cardiovascular disease, type 2 diabetes) and all-cause mortality has been observed among adults [2]. Conversely, excessive screen time (e.g., television, video/computer games) is associated with poorer health outcomes across the lifespan, such as obesity among young children [3] and adults [4] as well as impaired psychosocial and cognitive development among young children [3]. Furthermore, physical activity and screen time habits formed at an early age may track overtime [5,6]. Therefore, early childhood is a critical period for establishing healthy habits for both current and later health. Active parents can serve as important role models for their children while maximizing their own health [7].

Despite the known benefits of a healthy active lifestyle, a large proportion of young Canadian children and their parents are not meeting recommended levels [8,9]. For instance, national surveillance data indicate that only 15% of Canadian 3- to 4-year-olds meet both the physical activity and sedentary behavior guidelines [8]. In addition, only 43% of 0- to 4-year olds from Kingston, Canada meet the screen time recommendations that are part of the Canadian Sedentary Behavior Guidelines for the Early Years [10]. Similarly, only 8% of mothers and 10% of Canadian fathers with children <6 years old met recommendations of 150 minutes per week of moderate-to-vigorous intensity physical activity [9]. In fact, Canadian mothers and fathers with a child <6 years old are the only group of parents less likely (odds ratio = 0.31 for mothers; odds ratio = 0.34 for fathers) to meet physical activity recommendations when compared with adults with no dependent children [9]. While sedentary behavior guidelines do not currently exist for adults, data from various population-base studies indicate that two-thirds of adults accumulate more than 2 hours per day of recreational screen time [11].

To develop evidence-based interventions to promote healthy active lifestyles in young children and their parents, a greater understanding is needed of the correlates of physical activity and screen time in these dyads. Features of the physical environment in neighborhoods where families live have the potential to encourage or discourage the physical activity and screen time behaviors of young children and their parents [12]. There are a growing number of studies examining the relationship between the physical environment and adult’s physical activity [12-15]. To date, findings have been relatively inconsistent but a recent review indicates availability/connectivity of trails and accessibility/convenience of recreation facilities show promise [13]. However, it is unclear if features of the physical environment have similar or different influences on parents with young children, given the unique time and energy demands of child care. Furthermore, little is known regarding the influence of the physical environment on the physical activity of young children [16] and on the screen time of young children and their parents [17-19].

To our knowledge no study has examined the influence of features of the physical environment on physical activity and screen time of young child–parent dyads. While it is clear young children have little autonomy from their parents [20], it is unclear whether features of the physical environment have similar or different influences on behaviors in these different age groups. This has important implications on the strategies used in future interventions and public health initiatives aiming to promote a healthy active lifestyle in young children and their parents.

Using previous qualitative and quantitative research, Pikora and colleagues have developed a conceptual framework for examining the influence of the neighborhood physical environment on physical activity [14]. The framework includes function (i.e., walking surface, streets, traffic, permeability), safety (i.e., personal, traffic), aesthetic (i.e., streetscape, views), and destination (i.e., facilities) physical environment features [14]. The purpose of this study was to use this framework as a guide to examine the associations between several features of the physical environment with physical activity and screen time among young children (≤5 years old) and their parents after taking into account several socio-demographic factors.

## Methods

### Participants

This study is based on the Healthy Living Habits in Pre-school Children study. Data were collected between May and September 2011 on children ≤5 years old and their parents. All participants resided in the Kingston, Frontenac, Lennox, and Addington Health Region in Ontario, Canada. Parents of pre-school children were recruited from licensed child care centers (46 of the total 60 centers participated) and public health/community programs (14 of the total 16 programs participated). Eligible parents received a package containing a questionnaire that had been pilot tested. Approximately 37% of parents returned the brief (15 minutes) questionnaire resulting in a total sample of 800. Ethics approval was obtained from the Queen’s University General Research Ethics Board. Consent was obtained from participating child care centers, public health/community programs, and parents.

### Neighborhoods

Using census dissemination areas, the city of Kingston is comprised of 45 distinct neighborhoods with common social, physical, and political attributes. Neighborhood-level measures were based on the neighborhood that each child–parent dyad lived in, which was determined by participant’s home address or postal code. For participants who only provided a postal code (n = 89), a point was placed on the geographic center of the postal code, which typically represents a street block, to determine the neighborhood they lived in [21].

### Physical environment measures

The selection of physical environment measures was based on previous literature on the relationship between the environment with physical activity and/or screen time in children, youth, or adults [13,16,18,22-25] and was guided by the conceptual model developed by Pikora and colleagues [14]. A combination of ArcGIS (ESRI’s ArcInfo 10) software with CanMaps® Streetfiles, CanMap® Route Logistics*,* CanMap® Parks and Recreation, Enhanced Points of Interest database (DMTI Spatial Inc., 2009), and Google Earth Street View were used to assess the environment features.

#### Function

##### Walkability

Walkability was assessed by five items at the neighborhood level using ArcGIS software with CanMap® Streetfiles, CanMap® Route Logistics, and Google Earth Street View. Average block length was calculated by dividing total road length by the number of true intersections (3- or 4-way intersections) [26]. Connected intersection ratio was calculated by dividing the number of true intersections by the number of total intersections (i.e., street intersections, cul-de-sacs and dead ends) [26]. Intersection density was calculated by dividing the number of true intersections by the total land area [26]. Residential area was calculated by dividing residential land area by total land area [24]. The sidewalks variable was calculated by dividing the road distance with a sidewalk on one or both sides by the total road distance [27]. Principal component analysis was conducted on the five items to create a summary walkability variable. One component with an eigenvalue of 3.4 emerged, explaining 68.2% of the total variance.

#### Safety

##### Road speed

Road speed was assessed by a single item at the neighborhood level using ArcGIS software with CanMap® Streetfiles, and CanMap® Route Logistics. It was calculated by dividing road length with low speeds (<61 km/h) by total road length. Road speed limits in residential areas in Canada are typically <60 km/hr, thus ≥61 km/hr was chosen to represent roads with high traffic speeds that tend to have greater traffic volumes.

#### Aesthetics

##### Streetscape

Streetscape was assessed with three items at the neighborhood level using ArcGIS software with CanMaps® Streetfiles and Google Earth Street View. The three items included conditions of buildings/grounds, graffiti, and litter, as explained in detail elsewhere [28]. These measures have good intra-rater reliability (r = 0.82 to 0.99), inter-rater reliability (r = 0.75 to 0.95), and validity (r = 0.65 to 0.91) [28]. Principal component analysis was conducted on the three items to create a summary aesthetics variable. One component with an eigenvalue of 1.9 emerged, explaining 61.7% of the total variance.

#### Facilities

##### Outdoor play/activity space

Outdoor play/activity space was assessed with three items at the neighborhood level using ArcGIS software with CanMap® Streetfiles, CanMap® Route Logistics*,* CanMap® Parks and Recreation, and Google Earth Street View. Cul-de-sac density was calculated by subtracting the number of true intersections from the total number of intersections and dividing by the total land area [29]. Open wooded area was calculated by dividing area devoted to wooded areas by total land area [24]. Parks/sports fields was calculated by dividing the area devoted to parks or sports fields by total land area [21]. Principal component analysis was conducted on these three items to create a summary outdoor play/activity space variable. One component with an eigenvalue of 1.7 emerged, explaining 56.8% of the total variance.

##### Recreation facilities

Recreation facilities were assessed by a single item at the neighborhood level using ArcGIS software and the Enhanced Points of Interest database. Recreation facilities included arenas, yoga/dance studios and halls, bowling centres, physical fitness facilities, public golf courses, and sports and recreation clubs. The item was calculated by dividing the total number of recreation facilities by the total land area [21,30].

##### Distance to closest park

The shortest distance from participants’ homes to the closest park, as travelled on the road network or via neighborhood trails/paths, was assessed for each child–parent dyad using ArcGIS software with CanMap® Streetfiles, CanMap® Route Logistics*,* CanMap® Parks and Recreation, and Google Earth Street View [24].

##### Yard space

Yard space at each child–parent dyad’s place of residence was assessed using ArcGIS software and Google Earth Street View. It was calculated by subtracting the building area (including detached buildings such as a shed/garage) from the parcel area [24]. For participants who only provided their postal code (n = 89), the average building size was subtracted from the average parcel area for the block pertaining to their postal code. Finally, for participants who lived in apartment buildings (generally represented by one postal code), green areas around the building were calculated as yard space.

### Dependent variables

#### Physical activity in children

The typical amount of physical activity children participated in was assessed with three items adopted from Statistic Canada’s National Longitudinal Study of Children and Youth (NLSCY) [31]. Two items were specific to frequency of organized and unorganized activities. There were five response options for each item ranging from “Most days” to “Almost never”. The third item was specific to duration. There were 4 response options ranging from “1 to 15 minutes” to “More than 1 hour”. Physical activity was determined by multiplying the duration item by the average of the two frequency items. A previous validation study reported that a brief parental questionnaire used to measure the physical activity of young children, similar to that used in the present study, had a significant albeit moderate correlation (r = 0.33) with physical activity measured using accelerometers [32].

#### Screen time in children

The amount of time children spent watching television and playing video/computer games was assessed with two items adopted from Statistic Canada’s NLSCY [31]. Both items were rated on a 7-point scale for weekday and weekend use ranging from ‘none’ to ‘≥3 hours/day’. Weighted means of weekday and weekend use were calculated and screen time was determined by adding television and video/computer games use. Parental report is commonly used in the literature to assess television viewing among young children [33]. A previous validation study reported that responses to a brief parental questionnaire on child television viewing time, similar to the questions used in the present study, was significantly correlated (r = 0.60) with television viewing measured by a parental diary [34].

#### Physical activity in parents

Physical activity in parents during a typical week was assessed using the Godin Leisure Time Exercise Questionnaire [35]. Parents reported the number of times per week they did strenuous, moderate, and mild exercise for more than 15 minutes in their free time. A physical activity score was created by multiplying strenuous times per week by 9, moderate times per week by 5, and mild times per week by 3 [35]. The questionnaire has previously shown to have good test- re-test reliability (r = 0.74) and low to moderate validity (r = 0.24), when V0_2_max was used as the criterion [35].

#### Screen time in parents

The typical weekly time parents spent watching television/videos/DVDs, using a computer (not for work/school), and playing video games was assessed with the three items adopted from Statistics Canada’s Canadian Community Health Survey [36]. All items were rated on an 8-point scale ranging from ‘none’ to ‘>20 hours’. Screen time was determined by adding television, computer, and video game use.

### Socio-demographic characteristics

The age and sex of the child, sex and education of the parent, child care status (yes or no), and family structure (two-parent or single-parent home) were assessed with the parental questionnaire. Parents’ education was measured by asking the parent completing the questionnaire what their highest complete grade or level of education was. There were six options ranging from “no schooling” to “graduate university” [31]. Neighborhood socioeconomic status (SES) was assessed with three items including education (percentage of adult residents with less than a high school education), income (average employment income), and unemployment rate, which was measured using 2006 Canadian census data in PCensus for MapPoint through ArcGIS software. Principal component analysis was used to create a continuous summary neighborhood SES score.

### Statistical analysis

Analyses were completed using SAS version 9.2 (SAS Institute Inc., Cary, NC). The physical activity and screen time variables for both children and parents were positively skewed so they were square root transformed for regression analyses. The MIXED procedure was used to account for the hierarchical and clustered nature of the data. To address the main objective of the paper, bivariate multilevel linear regression models were initially run to examine the associations between physical environment and the socio-demographic variables with the four outcomes (physical activity and screen time for children and parents). All variables that had a statistically significant (*P* < 0.05) association with the outcome in the bivariate models were then entered into a multivariate model. A sample size of *n* = 511 was deemed sufficient to detect a medium effect (i.e., r = 0.30) at an alpha level of 0.05 and 80% power [37].

## Results

A total of 62 participants were excluded because they were missing the birth date or sex of the child or physical activity data. Another 15 participants were excluded because they lived in rural neighborhoods where Google Earth Street View was not available. Finally, a further 212 participants were excluded because they lived outside of the 45 Kingston neighborhoods where extensive GIS data was not available. In total, 511 child–parent dyads from 37 neighborhoods were included in the analyses. There were no significant differences in the physical activity and screen time variables between excluded and included participants.

Participant characteristics of the 511 children and parents are in Table 1. Approximately 55% of the children were male, 4% were less than <1 years old, 61% were aged 1–3 years, and 35% were aged 4–5 years. Children participated in a median of 57 min/day of screen time and 23 min/day of physical activity. Parents participated in a median of 81 min/day of screen time, light physical activity 4 times/week, moderate physical activity 2 times/week, and strenuous physical activity 1 time/week. Characteristics of the 37 neighborhoods are in Table 2. Of note, median income was $72,217, a median 62% of roads had sidewalks, and a median 50% and 6% of total land area was residential area and parks/sports fields, respectively.

**Table 1:** Individual-level characteristics

	***Total *** **(N = 511)**
Child sex (%)	
Male	55.2
Female	44.8
Parent sex (%)	
Male	8.6
Female	91.4
Child age (%)	
Infants (< 1 years old)	4.3
Toddlers (1–3 years old)	60.5
Preschoolers (4–5 years old)	35.2
Child care status	
Yes	83.8
No	16.2
Family structure (%)	
Two-parent home	70.8
Single-parent home	29.2
Parental Education (%)	
Elementary (Grades 1–8)	1.2
High school (Grades 9–12)	14.1
Community/technical college	32.1
University bachelor degree	26.2
University graduate degree	26.4
Distance to closest park (km)	0.2 (0.1, 0.4)
Yard space at home (m^2^)	467 (288, 885)
Child physical activity (min/day)	22.8 (13.0, 30.0)
Child screen time (min/day)	57.4 (28.4, 106.6)
Parent physical activity	
Light (times/week)	4 (2, 7)
Moderate (times/week)	2 (0, 4)
Strenuous (times/week)	1 (0, 3)
Parent screen time (min/day)	81.4 (42.9, 137.1)

Data presented as median (inter-quartile range) or %.

**Table 2:** Neighborhood-level characteristics

	***Total *** **(*N*** **= 37 neighborhoods)**
Neighborhood SES	
Education (% < high school)	7.3 (5.2, 13.8)
Average income ($ CAD)	72,217 (53,806, 90,488)
Unemployment (%)	3.9 (2.8, 7.7)
Walkability	
Average block length (km)	0.3 (0.3, 0.4)
Connected intersection ratio (% of true intersections)	80.4 (76.6, 85.7)
Intersection density (N per km^2^)	35.5 (11.4, 42.4)
Residential area (% of land area)	50.4 (20.5, 74.8)
Sidewalks (% of roads with sidewalks)	61.5 (44.1, 78.1)
Road speed (% roads <61 km/h)	99.7 (84.2, 100.0)
Streetscape	
Conditions of buildings/grounds (1–4 point scale)	2.0 (2.0, 2.1)
Graffiti (1–4 point scale)	1.0 (1.0, 1.2)
Litter (1–5 point scale)	1.9 (1.5, 2.3)
Outdoor play/activity space	
Cul-de-sac density (N per km^2^)	6.5 (3.1, 9.0)
Open wooded area (% of land area)	2.3 (0, 12.8)
Parks/sports fields (% of land area)	6.2 (2.8, 8.3)
Recreation facilities (N per km^2^)	0.4 (0, 1.6)

Data presented as median (inter-quartile range).

The associations between physical environment and socio-demographic variables with the child and parent physical activity levels are in Table 3. In the multivariate model, none of the physical environment variables were associated with physical activity in the children or parents. However, associations were observed with socio-demographic factors. For children, not attending child care was associated with lower physical activity levels while being older and having a higher family SES was associated with higher physical activity levels. For parents, being female was associated with lower physical activity levels.

**Table 3:** The relationship between individual- and neighborhood-level variables and physical activity

	**Bivariate model**	**Multivariate model**
	***β *** **(95% CI)**	***β *** **(95% CI)**
**Child physical activity†**		
*Socio-demographic*		
Child age (months)	0.02 (0.02, 0.03)*	0.02 (0.02, 0.03)*
Child sex	-0.10 (-0.35, 0.15)	-
Child care status	-0.71 (-1.05, -0.38)*	-0.34 (-0.68, -0.00)*
Family structure	-0.16 (-0.48, 0.16)	-
Parental education	0.19 (0.07, 0.31)*	0.14 (0.02, 0.26)*
Neighborhood SES (z-score)	0.21 (-0.07, 0.35)*	0.09 (-0.06, 0.23)
*Functional*		
Walkability (z-score)	-0.09 (-0.27, 0.09)	-
*Safety*		
Road speed (%)	-0.34 (-0.90, 0.21)	-
*Aesthetic*		
Streetscape (z-score)	0.05 (-0.10, 0.21)	-
*Destination*		
Outdoor play/activity space (z-score)	-0.08 (-0.25, 0.09)	-
Recreation facilities (N per km^2^)	-0.01 (-0.07, 0.06)	-
Distance to closest park (km)	0.01 (-0.01, 0.02)	-
Yard space at home (100 m^2^)	-0.01 (-0.01, 0.00)	-
**Parent physical activity†**		
*Socio-demographic*		
Child age (months)	0.03 (0.02, 0.03)*	0.00 (-0.01, 0.02)
Child sex	-0.34 (-0.82, 0.16)	**-**
Parent sex	-1.35 (0.49, 2.25)*	-1.35 (0.49, 2.21)*
Child care status	0.33 (-0.43, 0.88)	**-**
Family structure	0.14 (-0.48, 0.76)	-
Parental education	-0.17 (-0.40, 0.06)	-
Neighborhood SES (z-score)	-0.11 (-0.34, 0.13)	-
*Functional*		
Walkability (z-score)	0.18 (-0.09, 0.45)	**-**
*Safety*		
Road speed (%)	0.05 (-0.78, 0.88)	-
*Aesthetic*		
Streetscape (z-score)	-0.13 (-0.34, 0.09)	-
*Destination*		
Outdoor play/activity space (z-score)	0.03 (-0.22, 0.27)	**-**
Recreation facilities (N per km^2^)	0.06 (-0.05, 0.17)	**-**
Distance to closest park (km)	-0.01 (-0.04, 0.03)	-
Yard space at home (100 m^2^)	-0.00 (-0.02, 0.01)	-

β (95% CI) = unstandardized regression coefficients and 95% confidence intervals; †Square-root transformed.

Child sex: 0 = male, 1 = female; parent sex: 0 = male, 1 = female; child care status: 0 = yes, 1 = no; family structure: 0 = two-parent family, 1 = one-parent family, ST = screen time; PA = physical activity; SES = socioeconomic status. Each variable in the multivariate models are adjusted for all other variables listed in that model; * ***P*** **< 0.05**.

The associations between physical environment and socio-demographic variables with child and parent screen time levels are in Table 4. In the multivariate model, none of the physical environment variables were associated with screen time in children. However, being older was associated with higher levels of screen time and having higher family and neighborhood SES were associated with lower levels of screen time. For parents, higher outdoor play/activity space (e.g., parks, wooded areas) was associated with higher screen time levels and having a higher neighborhood SES was associated with lower screen time levels.

**Table 4:** The relationship between individual- and neighborhood-level variables and screen time

	**Bivariate model**	**Multivariate model**
	**β (95% CI)**	**β (95% CI)**
**Child screen time†**		
*Socio-demographic*		
Child age (months)	0.10 (0.08, 0.11)*	0.10 (0.08, 0.11)*
Child sex	-0.76 (-1.39, -0.13)*	-0.44 (-0.99, 0.12)
Child care status	-0.43 (-1.30, 0.43)	-
Family structure	0.22 (-0.49, 0.93)	-
Parental education	-0.80 (-1.10, -0.51)*	-0.76 (-1.03, -0.48)*
Neighborhood SES (z-score)	-0.42 (-0.73, -0.12)*	-0.39 (-0.67, -0.11)*
*Functional*		
Walkability (z-score)	0.01 (-0.38, 0.41)	-
*Safety*		
Road speed (%)	0.63 (-0.59, 1.86)	-
*Aesthetic*		
Streetscape (z-score)	-0.00 (-0.32, 0.32)	-
*Destination*		
Outdoor play/activity space (z-score)	0.04 (-0.32, 0.40)	-
Recreation facilities (N per km^2^)	0.02 (-0.13, 0.17)	-
Distance to closest park (km)	-0.04 (-0.09, 0.01)	**-**
Yard space at home (100 m^2^)	0.02 (-0.002, 0.03)	**-**
**Parent screen time†**		
*Socio-demographic*		
Child age (months)	0.01 (-0.01, 0.03)	-
Child sex	0.27 (-0.34, 0.91)	-
Parent sex	0.69 (-0.45, 1.83)	-
Child care status	0.66 (-0.22, 1.53)	-
Family structure	-0.02 (-0.85, 0.81)	-
Parental education	-0.30 (-0.62, 0.01)	**-**
Neighborhood SES (z-score)	-0.65 (-0.96, -0.34)*	-0.59 (-0.89, -0.28)*
*Functional*		
Walkability (z-score)	0.04 (-0.02, 0.83)	**-**
*Safety*		
Road speed (%)	1.27 (-0.03, 2.58)	**-**
*Aesthetic*		
Streetscape (z-score)	0.05 (-0.31, 0.42)	**-**
*Destination*		
Outdoor play/activity space (z-score)	0.47 (0.10, 0.85)*	0.38 (0.06, 0.70)*
Recreation facilities (N per km^2^)	0.14 (-0.02, 0.30)	**-**
Distance to closest park (km)	-0.03 (-0.08, 0.02)	-
Yard space at home (100 m^2^)	0.01 (-0.01, 0.03)	-

β (95% CI) = unstandardized regression coefficients and 95% confidence intervals; †Square-root transformed.

Child sex: 0 = male, 1 = female; parent sex: 0 = male, 1 = female; child care status: 0 = yes, 1 = no; family structure: 0 = two-parent family, 1 = one-parent family, ST = screen time; PA = physical activity; SES = socioeconomic status. Each variable in the multivariate models are adjusted for all other variables listed in that model; * ***P*** **< 0.05**.

## Discussion

This study examined independent associations between features of the physical environment on physical activity and screen time among a sample of children ≤5 years old and their parents. Only one independent association was observed with the physical environment features and the association was in the opposite direction to what was expected. Specifically, higher outdoor play/activity space was associated with a higher screen time levels in parents. For physical activity, child age, child care status, and family SES were independent correlates for children while sex was an independent correlate for parents. For screen time, child age and family SES were independent correlates for children while neighborhood SES was an independent correlate for parents.

There is a growing body of literature examining the influence of several physical environment variables on physical activity among adults [13,15,25,38]. Pikora and colleagues developed a conceptual framework that categorizes the different physical environment variables into four main features (function, safety, aesthetic, destination). A narrative review identified positive associations between availability, accessibility and convenience of destinations, functionality of the neighborhood, and aesthetics with adult physical activity [25]. However, a recent systematic review only identified consistent associations with a few physical environment variables [13]. More specifically, connectivity of trails was identified as a consistent predictor of active commuting and accessibility/convenience of recreation facilities and availability of trails were identified as possible predictors of moderate-to vigorous-intensity physical activity among adults [13]. In line with the present study, inconsistent or null associations were observed for a number of other variables (e.g., availability of sidewalks, traffic volume, street lights, hills) [13]. Less research has examined the relationship between the physical environment and physical activity among young children. A recent narrative literature review reported that time spent outdoors/in play spaces is the only feature of the physical environment that is consistently associated with physical activity within pre-school children (aged 2–5 years) [16].

While some associations have been observed between the physical environment and adult physical activity, it is important to note that the present study focused on a sub-group of adults who have young children. This sub-group has been found to be less physically active compared to adults without young children [9,39,40]. Additional physical activity barriers experienced by parents with young children, such as the time and energy demands of child care, may explain this trend [9,39]. Thus, in comparison to the general adult population, the physical environment may be less important compared to other correlates of physical activity participation (e.g., access to social support) [39].

In comparison to physical activity, little is known about the relationship between the physical environment and screen time among young children or adults [17,18]. For example, no physical environment predictors of screen time were identified in two recent reviews among young children [18,19]. One of the few studies to examine this relationship in adults found neighborhood walkability was negatively associated with television viewing among women but not men in a small sample of Australian adults [17]. Conversely, outdoor play/activity space was positively associated with screen time among parents in the present study. It is unclear why more outdoor play/activity space in a neighborhood would be related to more screen time in adults. Perhaps this neighborhood feature is not relevant in providing alternative screen time activities to parents.

While the physical environment had little influence on physical activity and screen time behaviors of young children and their parents in the present study, several socio-demographic factors were of importance. Many of the associations observed were consistent with previous findings in the literature. For instance, sex (being female) was reported as a consistent negative correlate of physical activity in a systematic review among adults [41]. Similarly, two recent reviews identified age as a consistent positive correlate and SES as a consistent negative correlate of screen time in young children [18,19]. Furthermore, a negative relationship was observed between neighborhood SES and screen time in another large sample of young Canadian children [42]. The findings of this study suggest that socio-demographic factors, including social environment factors, may be stronger correlates of physical activity and screen time behaviors of young children and their parents compared to features of the physical environment. To our knowledge this is the first study to examine these associations in young child–parent dyads. Therefore, due to the overall dearth of information, future research is needed to confirm and build upon these findings.

Study strengths include the objective measures of the environment and the large sample size, which allowed for the examination of multiple environment measures. A main limitation is the cross-sectional design, which limits the assessment of causality. Further, the physical activity and screen time measures were either self- or parent-reported. While subjective measures have frequently been used in the environment and physical activity literature [13], the information bias associated with these measures may result in an underestimation of the true associations [43]. Additionally, the sample was not representative due to the low response rate and the fact that the main source of recruitment was licensed child care centers. Only 15% of young children in the health region attend these centres [44], and their high attendance cost is a likely barrier. Therefore, caution should be taken when generalizing the findings due to the relatively high socioeconomic status of our sample.

## Conclusions

The findings from the present study suggest that socio-demographic factors, including social environment factors, may be more important targets than features of the physical environment for future interventions aiming to promote a healthy active lifestyle in young children and their parents. Due to the dearth of information, future research is needed in young child–parent dyads to confirm and build upon these findings.

## Competing interest

The authors declare that they have no competing interests.

## Authors’ contributions

VC assisted with the design of the study, collected the questionnaire data, led the statistical analysis, and wrote the initial draft of the manuscript. AR collected the environment data and revised the manuscript for important intellectual content. IJ assisted with the design of the study, provided insight and guidance on the statistical analysis, and revised the manuscript for important intellectual content. All authors approved the final version of the manuscript.

## Pre-publication history

The pre-publication history for this paper can be accessed here:

http://www.biomedcentral.com/1471-2458/14/61/prepub
